# C677T polymorphism increases the risk of early spontaneous abortion

**DOI:** 10.1007/s10815-019-01500-2

**Published:** 2019-06-17

**Authors:** Yongjie Liu, Fan Zhang, Liang Dai

**Affiliations:** Department of Reproductive Center, Yinchuan Maternal and Child Health Hospital, No. 56, Culture West Street, Xingqing District, Yinchuan, 750001 Ningxia China

**Keywords:** Male, MTHFR C677T polymorphism, Early spontaneous abortion

## Abstract

**Purpose:**

To investigate the relationship of the methylenetetrahydrofolate reductase (MTHFR) C677T polymorphism in male population having spouses with early spontaneous abortion.

**Methods:**

A total of 345 males whose spouses had at least one early spontaneous abortion were included in the study group, and 145 males who planned to have a second child were included in the control group. Semen was collected in a sterile cup by masturbation. After liquefaction, the sperm concentration and forward motility sperm rate (PR) were obtained. The genomic DNA was extracted from peripheral vein, followed by MTHFR C677T polymorphism detection through PCR-gold magnetic nanoparticle chromatography.

**Results:**

The numbers of alleles and genotypes of MTHFR in the case group were 303 (C), 387 (T), 64 (CC), 175 (CT), and 106 (TT) cases, respectively. The numbers of allele and genotype of MTHFR in the control group were 167 (C), 123 (T), 145 (CC), 65 (CT), and 29 (TT) cases. There were significant differences in the distribution frequency of genotypes (*χ*^2^ = 17.005, *P* = 0.000) and alleles (*χ*^2^ = 15.295, *P* = 0.000) between the two groups. For cases with more spontaneous abortion, more cases had CT and TT phenotypes. Participants with genotype CT had the highest sperm concentration and PR in both groups (*P* < 0.05).

**Conclusions:**

MTHFR could affect sperm DNA integrity through affecting DNA methylation, which led to an increase in the rate of early spontaneous abortion in spouses.

**Electronic supplementary material:**

The online version of this article (10.1007/s10815-019-01500-2) contains supplementary material, which is available to authorized users.

## Introduction

Spontaneous abortion is one of the prevalent negative reproductive outcomes among women around the world, which is a great challenge faced by maternal health promotion [1]. Spontaneous abortion is defined as the loss of a clinically recognized pregnancy that occurs before 20 weeks of gestational age [2]. According to the time of pregnancy termination, spontaneous abortion can be classified as early abortion (< 12 weeks) and late abortion (≥ 12 weeks) [3]. Multiple pathological factors are related to early spontaneous abortion, such as abnormal embryo chromosomes, abnormal reproductive organs of pregnant women, and infection of reproductive tract [4]. However, there are limited male reproductive examinations for early spontaneous abortion analysis.

Methylenetetrahydrofolate reductase (*MTHFR*) is a key enzyme of folate/homocysteine pathway [5]. *MTHFR* play a critical role in the process of reproduction. The adverse effects of MTHFR deficiency on spermatogenesis maybe mediated partly by alterations in the transmethylation pathway, suggesting that betaine supplementation may provide a way to bypass MTHFR deficiency and its adverse effects on spermatogenesis by maintaining normal methylation levels within male germ cells [6]. It has been reported that *MTHFR* gene polymorphism can affect sperm quality [7]. Homozygosity for *MTHFR* C677T (late and early-late) and A1298C (early-late) was the risk factor for idiopathic recurrent pregnancy losses and was not related to total homocysteine levels [8].

In this study, the *MTHFR* gene C677T polymorphism of males whose spouses had been through early spontaneous abortion was detected, the relationship between *MTHFR* C677T polymorphism and early spontaneous abortion was analyzed, and the aim was to find possible measures to intervene early spontaneous abortion from male perspective.

## Materials and methods

### Study subjects

A total of 345 males whose spouses had at least one early spontaneous abortion were included in the case group, and 145 males planned to have a second child were included in the control group. All subjects in the case group visited the reproductive center of our hospital from January 2015 to August 2017. All subjects were asked about their medical history and underwent the physical examination of genital system. Patients with diseases such as chromosomal abnormality, cryptorchidism, varicocele, and reproductive system infection were excluded. The age of subjects was in the range of 21 to 42 years (30.29 ± 4.85). This study was approved by the Ethics Committee of our hospital. Before study, informed consent was obtained from each subject.

### Semen analysis

Semen was collected through masturbation after 3–5 days of abstinence, followed by liquidation at 36 °C. Sperm concentration and forward motility sperm rate (PR) were obtained by using a computer-assisted sperm analysis system (Beijing, Tsinghua Tongfang MX7.8.5-T) according to the WHO laboratory test manual for human semen (5th edition) [9].

### MTHFR C677T detection

The C677T mutation was analyzed by PCR-gold magnetic nanoparticle chromatography (Xi’an GoldMag Nanobiotech Co., Ltd.) with Applied Biosystems 2720 Thermal Cycler.

### Statistical analysis

Data were statistically evaluated using SPSS statistics v.17.0. The distribution of the allele of the MTHFR gene C677T between the two groups was compared. The difference of sperm concentration and PR of subjects in the two groups was analyzed. *F* test was used for the measurement data of multiple groups, *t* test was used for the comparison of the two groups, and *chi*-square test was used for comparing counting data. Continuous data were presented as the mean ± SD. *P* < 0.05 was considered to be statistically significant.

## Results

### Basic information of subjects

In the case group, the age of 345 males ranged (mean ± SD) from 21 to 42 (30.19 ± 4.46) years old, and the average BMI was 24.39 ± 3.84 kg/m^2^. A total of 106 cases had 1 time of spontaneous abortion, 125 cases had 2 times, and 114 cases had more than 2 times of spontaneous abortion. In the control group, the age of 145 males ranged (mean ± SD) from 23 to 40 (30.89 ± 5.39) years old, and the average BMI was 24.27 ± 3.73 kg/m^2^.

### Distribution of genotypes and alleles of MTHFR C677T

The genotyping results were shown in Table 1. In the case group, 64 cases had the genotype of CC, 175 cases had the genotype of CT, and 106 cases had the genotype of TT. In the control group, 51 cases had the genotype of CC, 65 cases had the genotype of CT, and 29 cases had the genotype of TT. Significant less proportion of cases had CC genotype (*χ*^2^ = 17.005, *P* < 0.001). In patients with 2 spontaneous abortion, 32 cases (25.6%) had CC, 62 cases (49.6%) had CT, and 31 cases (24.8%) had TT, respectively (Supplemental Table 1). In patients with more than 2 spontaneous abortion, 15 cases (13.2%) had CC, 52 (45.6%) cases had CT, and 47 cases (41.2%) had TT, respectively. In patients with more spontaneous abortion, more cases had CT and TT. The analysis of allele distribution showed that there were 303 cases with C and 387 cases with T in the case group, while in the control group, allele C was found in 167 cases, and T was found in 123 cases. Significant less allele C was observed in the case group (*χ*^2^ = 15.295, *P* < 0.001).

**Table 1:** Distribution of genotypes and alleles of MTHFR gene C677T

Genotype/allele		Case group	Control group	OR (95% CI)	*χ* ^2^	*P*
Genotype	CC	64 (18.55)	51 (35.17)	1	17.005	< 0.001
	CT	175 (50.7)	65 (44.83)	1.637(1.223—2.192)		
	TT	106 (30.73)	29 (20)	2.064(1.409—3.025)		
Total		345 (100)	145 (100)			
Allele	C	303 (43.91)	167 (57.59)	1	15.295	< 0.001
	T	387 (56.09)	123 (42.41)	1.473(1.211—1.793)		
Total		690 (100)	290 (100)			

### Sperm concentration and PR between the two groups

In the case group, the sperm concentrations were (74.42 ± 55.46) × 10^6^/ml for CC, (52.76 ± 32.38) × 10^6^/ml for CT, and (96.48 ± 58.85) × 10^6^/ml for TT. The average sperm concentration was (78.79 ± 59.66) × 10^6^/ml. In the control group, the sperm concentrations were (42.58 ± 27.42) × 10^6^/ml for CC, (65.60 ± 30.77) × 10^6^/ml for CT, and (41.85 ± 34.49) × 10^6^/ml for TT. The average sperm concentration was (52.76 ± 32.38) × 10^6^/ml. The sperm concentration in the case group was significantly higher than that in the control group. In both groups, subjects with the genotype of CT showed the highest concentration of sperm, while those with the genotype of TT had the lowest sperm concentration (Table 2).

**Table 2:** Sperm concentration of different genotypes between the two groups

		*n*	Sperm concentration (× 10^6^/ml)	*F*/*t*	*P*	95% CI
Case group	CC	64	74.42 ± 55.46	20.442	< 0.001	60.56–88.27
	CT	175	96.48 ± 58.85^a^			87.70–105.26
	TT	106	52.23 ± 53.22^a,b^			41.98–62.48
Control group	CC	51	42.58 ± 27.42^a,b^	10.506	< 0.001	34.87–50.29
	CT	65	65.60 ± 30.77^b,d^			57.98–73.23
	TT	29	41.85 ± 34.49^a,b,e^			28.73–54.97
Total	Case group	345	78.79 ± 59.66	4.960	< 0.001	72.47–85.11
	Control group	145	52.76 ± 32.38			47.44–58.07

^a^Compared with the case group CC, *P* < 0.05

^b^Compared with the case group CT, *P* < 0.05

^c^Compared with the case group TT, *P* < 0.05

^d^Compared with the control group CC, *P* < 0.05

^e^Compared with the control group CT, *P* < 0.05

The sperm PR was 43.56 ± 18.58% in the case group and 37.84 ± 15.68% in the control group (*P* < 0.05). In the case group, the sperm PR were 39.15 ± 19.51% for CC, 48.12 ± 16.26% for CT, and 38.69 ± 19.86% for TT, respectively. The average sperm PR was 43.56 ± 18.58%. In the control group, the sperm PR were 30.66 ± 16.07% for CC, 44.31 ± 15.58% for CT, and 35.97 ± 7.38% for TT. The average sperm PR was 37.84 ± 15.68%. The sperm PR in the case group was significantly higher than in the control group. In both groups, subjects with the genotype of CT showed the highest sperm PR (Table 3).

**Table 3:** Sperm PR of different genotypes between the two groups

		n	Sperm PR (%)	*F*/*t*	*P*	95% CI
Case group	CC	64	39.15 ± 19.51	11.356	< 0.001	34.28–44.03
	CT	175	48.12 ± 16.26^a^			45.70–50.55
	TT	106	38.69 ± 19.86^b^			34.87–42.52
Control group	CC	51	30.66 ± 16.07^a,b,c^	12.919	< 0.001	26.14–35.18
	CT	65	44.31 ± 15.58^c,d^			40.45–48.17
	TT	29	35.97 ± 7.38b^e^			33.16–38.77
Total	Case group	345	43.56 ± 18.58	2.908	0.004	41.60–45.53
	Control group	145	37.84 ± 15.68			35.27–40.42

^a^Compared with the case group CC, *P* < 0.05

^b^Compared with the case group CT, *P* < 0.05

^c^Compared with the case group TT, *P* < 0.05

^d^Compared with the control group CC, *P* < 0.05

^e^Compared with the control group CT, *P* < 0.05

## Discussion

*MTHFR* gene encodes one of the key enzymes in folate metabolism and is located on chromosome 1 (1p36.3), which has 12 exons [10]. By taking part in methionine cycle, *MTHFR* can affect gene expression through DNA methylation. Dozens of mutations of *MTHFR* gene have been found so far, and more and more diseases related to new mutations continue to be discovered [11]. It has been reported that C677T polymorphism of *MTHFR* gene is closely related to atherosclerosis, hypertension, heart disease, birth defects, Alzheimer’s disease, and hormone metabolism [12], which have drawn great attention. Exogenous folic acid supplementation can reduce the risk of these diseases.

The 677th nucleotide mutation is the most common SNP for *MTHF*R, which provides three kinds of genotype of *MTHFR* namely CC (wild type), CT (mixed type), and TT (homozygous) [13]. In vitro experiments showed the decrease of heat resistance and activity of mutant MTHFR enzyme, which resulted in maternal toxicity and the abortion by affecting Hcy metabolism [14]. In fact, males and females have equal contribution for early spontaneous abortion. Cornet et al. showed that defective methylation linked to MTHFR might contribute to sperm pathogenesis via increased sperm nucleus decondensation index [15]. In this study, males whose spouses had at least one early spontaneous abortion and males planned to have a second child were included as study subjects. The results of *MTHFR* C677T genotyping showed that the proportion of subjects with the genotype of TT in the case group was significantly higher than in the control group, which was consistent with the report about the percentage of TT genotype for local men (17.8%, 32/180) [16] and women (21.44%, 131/611) [17]. Such high percentage of TT genotype in the case group deserves a great attention. *MTHFR* is involved in DNA methylation, which is one of the most important regulatory mechanisms during the formation of spermatozoa. The decrease of *MTHFR* activity can lead to the fragmentation rate of DNA and increased rate of spontaneous abortions [18]. Aarabi et al. found that folic acid supplementation in MTHFR 677TT genotype mice resulted in no changes in general health, sperm counts, or methylation of imprinted genes, suggesting folic acid might be of little help in these patients [19]. The effect of betaine supplement according to the genotyping of *MTHFR* on sperm DNA integrity and spontaneous abortion of spouse will be investigated in the future.

In present study, sperm concentration and PR were assessed in two groups. It was found that the sperm concentration and PR in the case group were significantly higher than those in the control group. The CT genotype in the case group was the highest. The results were consistent with the report of Ghorbian et al. [20], and they found that the sperm concentrations of the case group and the control group were (45.57 ± 57.67) × 10^6^/ml and (37.97 ± 25.93) × 10^6^/ml. But Wang et al. [21] had given different results; the sperm concentration and PR in the case group and the control group were (75.23 ± 48.08) × 10^6^/ml and 33.19 ± 16.40% and (93.03 ± 59.62) × 10^6^/ml and 50.79 ± 17.64%, respectively. The results from Absalan et al. [22] indicated no significant differences in sperm concentration and PR between the case group and the control group. However, all reports indicated that sperm concentration and PR in the case group and the control group were significantly higher than the normal standard. The sperm concentration and PR of males with early spontaneous abortion cannot be used in the evaluation, treatment, and prevention of early spontaneous abortion [23]; thus, DNA detection was necessary. Carlus et al. demonstrated that *MTHFR* C677T polymorphism might raise plasma Hcy level [24]; Hcy can cause an increase of oxygen free radicals and affect sperm quality, or cause arteriosclerosis and affect reproductive function. In this study, subjects with CT genotype had the highest percentage in both group, suggesting that *MTHFR*-mediated Hcy metabolism had a limited effect on the reproductive system, and it worked in a different way to the reproductive system compared with other systems. It may have a little effect on spermatogenesis maturity.

In our study, we observed that cases with more spontaneous abortion had a higher proportion of CT and TT. The compound heterozygotes 677CT and 1298 AC have elevated homocysteine [25]; thus, this population might be at a higher risk of abortion. These two SNPs were not tested in our study and that was one limitation of our work.

## Conclusion

In summary, the *MTHF*R C677T polymorphism had a little effect on sperm concentration and PR. However, C677T polymorphism could affect the integrity of sperm DNA by affecting the DNA methylation, which led to an increase in the rate of early spontaneous abortion. The C677T polymorphism of *MTHFR* gene should be screened before pre-pregnancy. Due to the small sample size, further study with large sample will be performed to verify the results of this study.

## Electronic supplementary material

ESM 1 (DOCX 14 kb)
