# Supplementary material for: C677T polymorphism increases the risk of early spontaneous abortion
Source: J Assist Reprod Genet. 2019 Jun 17;36(8):1737–41. doi: 10.1007/s10815-019-01500-2 (PMC6707973; doi:10.1007/s10815-019-01500-2)
Supplement: Supplementary file 1 — (DOCX 14 kb) [file 10815_2019_1500_MOESM1_ESM.docx]

**Supplemental Table 1.** Distribution of genotypes of MTHFR gene C677T in patients with ≥2 spontaneous abortion

| No. of spontaneous abortion |  | CC | CT | TT | total |
| --- | --- | --- | --- | --- | --- |
| 2 |  | 32 (25.6) | 62 (49.6) | 31 (24.8) | 125 |
| >2 |  | 15 (13.2) | 52 (45.6) | 47 (41.2) | 114 |
| Total |  | 64 | 175 | 106 |  |
